# Xenotransplantation: benefits and risks.

**DOI:** 10.3201/eid0707.017724

**Published:** 2001

**Authors:** L Chapman

**Affiliations:** Centers for Disease Control and Prevention, Atlanta, Georgia 30333, USA. lec3@cdc.gov


able organic carbon level, degree of pipe corrosion, and treatment/distribution system characteristics. Chloramine is considerably more effective than chlorine for controlling *Legionella* in biofilms, presumably because chloramine is more stable and thus less reactive than chlorine, allowing it to penetrate the biofilm more deeply.

An important factor in distribution system contamination and bacterial growth on biofilms is transient water pressure fluctuations that create pressure waves that pass through pipes in the distibution system. During the negative portion of the pressure wave, a substantial amount of contaminated water (>1 gal per minute) from the outside can be pulled into pipes through a small leak. This problem is aggravated when sewer lines are placed close to water pipes. Dr. LeChevallier stated that a number of waterborne disease outbreaks have been linked to distribution system deficiencies. Among the agents of nosocomial waterborne disease is MAC. This opportunistic bacterial pathogen lives in water, is resistant to water disinfection (much more so than *Giardia* cysts), and grows in pipe biofilms.

### Xenotransplantation: Benefits and Risks

**Louisa Chapman**
Centers for Disease Control and Prevention, Atlanta, Georgia, USA

During transitional periods new scientific understandings bring new questions. The pivotal issues in xenotransplantation concern biohazards. The U.S. Public Health Service defines xenotransplantation as ". . . any procedure that involves the transplantation, implantation, or infusion into a human recipient of either A) live cells, tissues, or organs from a nonhuman animal source or B) human body fluids, cells, tissues, or organs that have had ex vivo contact with live nonhuman animal cells, tissues, or organs."

Prior to 1990, xenotransplants were largely whole organs; recipients survived only days or weeks. However, in most recent xenotransplantation trials, immunoprotected porcine neurologic, pancreatic, and hepatic cells are used to treat degenerative neurologic disorders, diabetes, or hepatic failure. Increasingly, xenotransplantation products function for prolonged periods in recipients who survive months or years.

Xenotransplantation is a public health concern because it has the potential to infect human recipients with agents that do not ordinarily infect humans, thereby introducing new infections to humans. Therefore, xenotransplantation combines a potential benefit with a potential risk to humans that is presently unknown.

Xenogeneic infections belong to a larger category of "bioproduct-acquired" infections, an example of which is simian virus 40 (SV40). SV40, a polyomavirus, contaminated polio vaccine stocks in the 1950s. Investigations into whether SV40 infection is associated with an increased risk of cancer have been inconclusive. This lingering uncertainty about the long-term significance of apparently innocuous persistent human infection with nonhuman viruses underscores the potential for therapeutic use of bioproducts to have unintended consequences.

The PHS Guideline on Infectious Disease Issues in Xenotransplantation describes a system of safeguards built around two key concepts: pretransplant screening of some animal herds, source animals, and xenotransplantation products to minimize the risk of xenogeneic infections with recognized pathogens and posttransplant surveillance of recipients for previously unrecognized xenogeneic organisms.

Endogenous retroviruses exist as inactive proviral DNA in the germline of all mammals adequately studied to date. However, inactive genomic endogenous retroviruses can often express active virus capable of infecting human cell lines in vitro. Thus, xenotransplantation products contain benign genomic DNA that, on transfer into a human, may express infectious retrovirus capable of creating active, persistent infection. The importance of this infectious potential of animal tissue devoid of any identifiable exogenous microorganisms has been the subject of much concern and scientific inquiry over the past 5 years.

To date, limited studies on humans exposed to pig cells and tissues have produced no evidence of porcine endogenous retrovirus infection. However, the persistent presence of microchimeric xenogeneic pig cells in the human recipient confirms that even temporary exposure to xenotransplantation products may continuously expose humans to infectious agents contained within them. For this reason, it is argued that humans should not be exposed to xenotransplantation products containing infectious agents whose ability to infect, cause disease in, or be spread among humans is incompletely defined.

Xenotransplantation is a process that occurs under controlled circumstances; thus, measures can be implemented to minimize associated iatrogenic biohazards. Studies performed in the service of developing policies on xenotransplantation can model other approaches to science-based risk minimization used for other bioproducts.

Address for correspondence: Louisa Chapman, Centers for Disease Control and Prevention, 1600 Clifton Road, Mailstop G19, Atlanta, GA 30333, USA; fax: 404-639-0868; e-mail: lec3@cdc.gov

